# Overexpression of miR-126 Protects Hypoxic-Reoxygenation-Exposed HUVEC Cellular Injury through Regulating LRP6 Expression

**DOI:** 10.1155/2022/3647744

**Published:** 2022-01-17

**Authors:** Md Sayed Ali Sheikh, A. Almaeen, A. Alduraywish, Basil Mohammed Alomair, Umme Salma, Li Fei, T. L. Yang

**Affiliations:** ^1^Department of Internal Medicine, College of Medicine, Jouf University, Sakaka, Saudi Arabia; ^2^Department of Pathology, College of Medicine, Jouf University, Sakaka, Saudi Arabia; ^3^Department of Gynecology and Obstetrics, College of Medicine, Jouf University, Sakaka, Saudi Arabia; ^4^Department of Cardiology, Xiangya Hospital, Central South University, China

## Abstract

The aim of the study was to explore the clinical impact of circulatory miR-126 as a candidate for novel biomarker in patients with coronary artery disease (CAD) and its protective role against hypoxia/reoxygenation- (H/R-) exposed HUVEC cellular injury. A total of 278 subjects, which included 153 subjects with angiographically confirmed CAD, 70 unstable angina subjects, and 55 healthy individuals, along with 18-hour HR-induced HUVECs were recruited in this study. Plasma miR-126 levels were significantly downregulated in stable and unstable CAD patients as well as 18-hour HR-exposed HUVECs as compared with controls. Stable and unstable CAD subjects were significantly differentiated from healthy individuals with a predictive value of AUC 0.903 and 0.923, respectively. Moreover, peripheral circulatory miR-126 expressions in elderly (71-90 years) stable and unstable CAD patients were comparatively lower than younger (30-50 years) subjects. The caspase-3 activity, intracellular ROS concentrations, and cellular viabilities were evidently increased in 18-hour HR-exposed HUVECs than in normal cells (*P* < 0.001). On the contrary, mimic expressions of miR-126 prominently reduced caspase-3 activity and intracellular ROS levels and markedly enhanced HUVEC cellular viabilities (*P* < 0.001). LRP6 expressions were significantly elevated in HR-induced HUVECs, whereas overexpression of miR-126 remarkably decreased LRP6 expressions (*P* < 0.001). Plasma miR-126 could be used as a novel biomarker for early prediction of CAD subjects. Overexpression of miR-126 significantly improved HUVEC cellular viabilities by downregulation of LRP6 protein expression, suggesting a potential therapeutic target for CAD patients.

## 1. Introduction

Ischemic coronary artery disease (CAD) is a predominant cause of high morbidity and mortality among all the races in both genders all over the world. The CAD incidence rate is amenable for increase by approximately 20% over the next nine years. Early detection of major risk factors, prompt diagnosis, and proper management can significantly decline morbidity and mortality [1]. Globally, invasive coronary angiogram (CAG) has been widely used to confirm the diagnosis of atherosclerotic ischemic coronary heart disease, but it has some limitations in its availability and unsuitability for extremely senescent patients and cases of end-stage renal failure. Therefore, noninvasive universal clinical biomarkers are required by clinicians to diagnose all types of CAD subjects. In the 20^th^ century, advanced percutaneous transluminal coronary angioplasty (PTCA) has become the treatment of choice for the CAD patients, yet multivessel lesions and complex left main coronary occlusion are not manageable by such a treatment modality. As a result, it is necessary to discover newer molecular targets for the management of CAD patients. Coronary artery disease occurs mainly due to progressive chronic atherosclerotic inflammation in the coronary arteries. Several research groups have demonstrated that different atherosclerotic linked microRNAs (miR-217, miR-200c, miR-146a, miR-34a, miR-221, and miR-19b) have an essential role in terms of development and progression of coronary atherosclerosis [2–4].

It was demonstrated that altered miR-126 is implicated in endothelial dysfunction, generation of stable atherosclerotic plaque and subsequently erosion, rupture of atherosclerotic plaque, and formation of thrombus within coronary artery that leads to acute coronary syndrome through several inflammatory signaling pathways including MAPK, PDGF, IGF1, and VCAM-1 [3–5].

In fact, low-density lipoprotein receptor-related protein 6 (LRP6) is one of the members of the low-density lipoprotein receptor (LDLR) family and significantly regulates lipid metabolism via several Wnt/*β*-catenin signaling pathways and is critically involved in various cardiovascular diseases including coronary artery disease [6]. Moreover, accumulating evidence showed that LRP6 expression levels were highly increased in human atherosclerotic coronary arteries and linked with platelet-derived growth factor receptor *β* [7]. Xu et al. reported mutations of LRP6 were genetically associated with early onset of coronary artery disease [8].

Several research groups have demonstrated that plasma miR-126 levels were remarkably deregulated in both single and complex CAD lesions as well as in acute coronary syndrome [9, 10]. Yang et al. explored the protective role of miR-126 against hypoxia/reoxygenation- (H/R-) induced injury on human cardiac microvascular endothelial cells (HCMECs) by inhibiting inflammatory response through regulating PI3K/Akt/eNOS signaling pathway [11]. Moreover, Matilde et al.'s research group demonstrated that miR-126 prevents age-related endothelial senescence by targeting hypoxia-inducible factor-1*α* (HIF-1*α*), suggesting a new therapeutic modulation of age-associated coronary artery disease [12].

However, the relationship between miR-126 and LRP6 associated with coronary artery diseases and HR-exposed HUVECs are not fully explored. Therefore, the current study investigated the clinical significance of circulating miR-126 in CAD patients, and its protective effects against hypoxia/reoxygenation- (H/R-) induced HUVEC cellular injury and the underlying molecular mechanism linked with LRP6.

## 2. Materials and Methods

### 2.1. Human Participants

153 stable angina subjects, 75 unstable angina participants, and 55 healthy populations were selected from Cardiology Department and Health Center of Xiangya Hospital of Central South University between January 2016 and July 2017. CAD was confirmed by two coronary interventional experts through invasive coronary angiogram, at least one major coronary artery with fifty or more than fifty percent atherosclerotic stenosis in both male and female subjects. According to ACC/ESC clinical guidelines, CAD patients were subcategorized into either stable or unstable groups. Patients with cardiomyopathy, COPD with pulmonary hypertension, rheumatic valvular heart diseases, heart failure, cerebrovascular accident, and implanted coronary stent or coronary artery bypass graft (CABG) were not included in this study. Well-matched age and gender, healthy controls who were free from cardiovascular diseases, chronic kidney or liver diseases, chronic inflammatory conditions, and malignancy were recruited. This study was carried out by adhering to Declaration of Helsinki Human Research Protocols, from all the participants, written informed consents were obtained prior to study, and the Ethical Review Board of Xiangya Hospital approved this research. Peripheral 5 mL venous blood samples were obtained from all the study subjects in EDTA-coated tubes. Within 30 minutes, all the samples were centrifuged twice: at 3,000 revolutions for 15 min at 4°C and at 15,000 revolutions for 10 min at 4°C, respectively; then cell and debris-free pure plasma was transferred in EP tubes and afterward preserved at −80°C for RNA extraction.

### 2.2. Cell Culture, Establishment of Hypoxia/Reoxygenation (H/R), and Transfection of HUVEC

Human umbilical vein endothelial cells (HUVECs) were obtained from the Cell Institute of Chinese Academy of Health Sciences (Shanghai, China). The cells were cultured in 6-well plates with Dulbecco's Modified Eagle's Medium (DMEM) containing 10 percent (*v*/*v*) heat-inactivated fetal bovine serum (FBS) in a 95% air and 5% CO_2_ constant incubator at 37°C. For generation of hypoxic model, the HUVECs were subjected to 1% O_2_, 5% CO_2_, and 95% N_2_ in a modular incubator using glucose, sodium pyruvate, and serum-free DMEM for 12 hours. After that, the cells were placed to a normoxic incubator maintaining 5% CO_2_, 37°C temperature, and 95% air for 6 hours to enhance hypoxia/reoxygenation-induced HUVEC cellular injury.

Lipofectamine 2000 reagents at 3 mg/mL concentrations were used for the transfections of HUVECs following the manufacturer's protocols. Mimic-miR-126 (50 nmol/L mimic) was used to regulate the mimic expression of miR-126 in HUVECs, and 50 nmol/L mimic-negative control (NC), that is similar in nature to mimic, was served as an inner control.

### 2.3. Luciferase Reporter Activity Assays

The target gene of miR-126 was confirmed by Luciferase reporter gene analysis. miR-126 was predicted with the complementary sequence of 3′-UTR of LRP6 by Target scan (http://www.targetscan.org/), and 24-well plate HUVECs were transfected with Lipofectamine 2000, mimic-miR-126, and NC-miR-126 for 18 hours. The luciferase activities were demonstrated through Dual-Luciferase Reporter Assay kits by following the company's instructions (Beyotime, Jiangsu, China), and the values were normalized to Renilla, and the data were measured by fluorescent activity.

### 2.4. Measurement of ROS, Caspase-3, and Cellular Proliferation

After proper hypoxia/reoxygenation treatment, 5 *μ*M dichlorodihydrofluorescein diacetate (DCFH-DA) was added into 96-well black plate cultured HUVECs and incubated for 30 minutes at 37°C [DCFH-DA, Molecular Probes, Eugene, USA]. After that, cells were washed twice by PBS, and finally, intracellular ROS levels were detected at 490 nm wavelengths through a SpectraMax microplate reader (Molecular Devices, Sunnyvale, CA).

Caspase-3 activities were demonstrated from healthy and treated HUVECs by adding 100 *μ*L caspase-3 substrate (Ac-DEVD-pNA) (Beyotime, Shanghai, China) into 96-well plates, incubated for 2 hours at 37°C, and absorbance was measured at the 405 nm wavelength by microplate SpectraMax absorbance reader (Molecular Devices, Sunnyvale, CA).

CCK-8 reagents were used according to the manufacturer's protocols for the detection of cellular viabilities (Beyotime, Shanghai, China). The 10 *μ*L CCK-8 solution was added into each 96-well culture plate's normoxic and HR-exposed HUVECs and inoculated for 2 hours at 37°C; then, cellular growths were assessed by a microplate absorbance reader at the wavelength of 450 nm (Molecular Devices, Sunnyvale, CA, USA).

### 2.5. Extraction of RNA and Analysis of miRNA Expressions

Total pure RNAs were isolated from human plasma and normoxic and treated HUVECs by using the TRIzol reagent (Invitrogen, CA, USA) following the company's guidelines. The primers of miR-126, mimic-miR-126, NC-miR-126, and inner control miR-156a were obtained from RiboBio (Guangzhou, China). LRP6 and endogenous control *β*-actin primers were collected from Sangon Biotech (Shanghai, China). Expression of miR-126 and LRP6 was determined through quantitative real-time polymerase chain reaction (qRT-PCR) technique by using Master Mix SYBR Green PCR reagents. In our previous studies, the measurement of miRNAs' expressions via qRT-PCR protocols was explained in details [13, 14].

### 2.6. Statistical Data Analysis

Statistical version 21 software (SPSS 21.0) was applied for the analysis of the data. For continuous variation, an independent-sample *t-*test, the Mann–Whitney, and one-way ANOVA were performed between groups. Among groups for categorical variation, the chi-square (*χ*^2^) and Fischer's exact tests were used. The clinical impact of plasma miR-126 as a biomarker was evaluated with Receiver Operating Characteristic (ROC) curve. A value of *P* < 0.05 indicated statistically significant results.

## 3. Results

### 3.1. Baseline and Clinical Characteristics of the Study Groups

The demographic general characteristics of the healthy, stable, and unstable participants in this study are detailed in Table 1. Among 153 patients with stable angina cases, 85 were males and 68 were females and their ages ranged between 30 and 90 years (65.1 ± 12.4); within the 70 unstable angina patients, 43 were males and 27 were females, and their ages ranged from 30 to 90 years (68.5 ± 14.8), and 55 healthy volunteers (30 males and 25 females), aged between 30 and 90 years (57.2 ± 11.3), were enrolled in the study, as well. Family history of coronary heart disease and hs-CRP levels were evidently higher in CAD (either stable or unstable angina cases) than control healthy individuals (*P* < 0.001). However, other variables such lipid profiles, AST, ALT, blood pressure, heart rate, glucose concentrations, cardiac function, smoking status, essential hypertension, and creatinine levels among healthy, stable, and unstable participants were compared but appeared statistically insignificant.

### 3.2. Expression of Plasma miR-126 in Coronary Artery Disease Patients and Hypoxia/Reoxygenation HUVEC Cells

**Figure 1:**
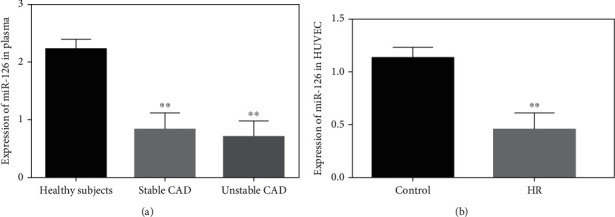
Circulatory miR-126 expression in CAD and HUVECs. (a) miR-126 concentrations between stable, unstable, and healthy individuals. (b) The expression levels of miR-126 within control and 18-hour hypoxia/reoxygenation HUVECs; miR-156a was used as an inner control. ^∗∗^*P* < 0.001.

Plasma miR-126 expression levels were significantly downregulated in stable coronary artery disease and unstable coronary artery disease patients by 2.7 (0.822 ± 0.29) and 3.2 (0.695 ± 0.29) folds, respectively, as compared with healthy participants (2.22 ± 0.67) (*P* < 0.001) (Figure 1(a)). Circulating plasma miR-126 concentrations were lower in unstable CAD patients than in stable CAD patients but statistically insignificant. Moreover, expressions of miR-126 were prominently downregulated in 18-hour hypoxia/reoxygenation- (H/R-) exposed HUVECs by 2.5 (1.130 ± 0.10) folds than normally incubated HUVECs (0.45 ± 0.16) (*p* < 0.001) (Figure 1(b)).

### 3.3. Relationship of miR-126 Expression with Aging and Gender

**Figure 2:**
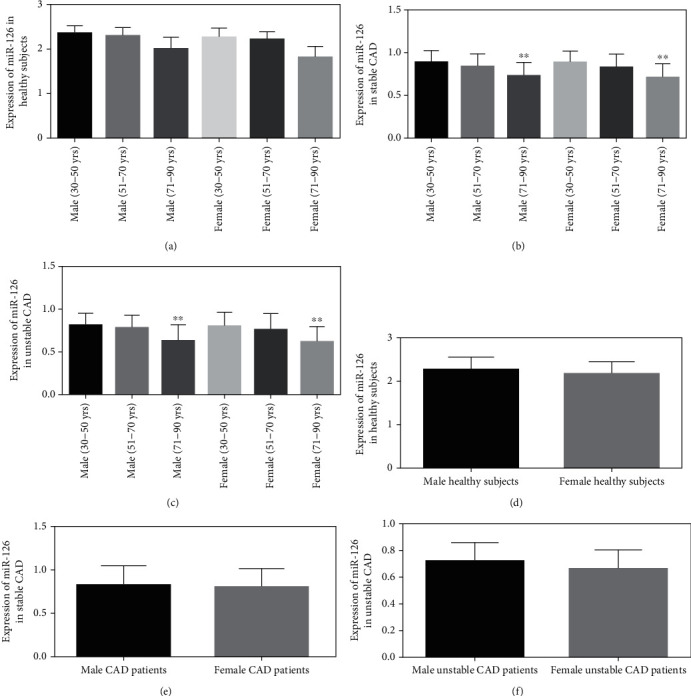
Expression patterns of circulatory miR-126 among different age groups in both male female subjects. (a) Plasma miR-126 expression among healthy subjects. (b) Circulatory miR-126 concentrations in stable CAD subjects. (c) Expression of plasma miR-126 in unstable angina patients. (d) Healthy male versus healthy female subjects' miR-126 levels. (e) Male versus female CAD subjects' plasma miR-126. (f) The level of miR-126 among unstable male and female CAD patients. ^∗∗^*P* < 0.001.

Circulating miR-126 expressions in healthy male and female (71-90 yrs) subjects were comparatively lower than younger male and female healthy participants in the age groups of 30-50 yrs and 51-70 yrs (*P* > 0.05) (Figure 2(a)). In either stable or unstable CAD subjects of both genders, circulating miR-126 levels in the extremely aged group (71-90 yrs) were significantly lower compared to those in younger male and female (30-50 yrs and 51-70 yrs) subjects (*P* < 0.001) (Figures 2(b) and 2(c)). These results suggested that underexpression of circulating miR-126 has strong correlation with geriatric people. However, the miR-126 expressions among the 30-50 yrs and 51-70 yrs groups in stable and unstable CAD patients of either sex were not significantly different. It was noted that the expression levels of miR-126 were the lowest in unstable angina cases followed by stable and healthy subjects. Gender wise, no significant variation in plasma miR-126 concentrations across all study groups (i.e., healthy, stable, and unstable angina groups) was discernible (*P* > 0.05) (Figures 2(d)–2(f)).

### 3.4. Clinical Significance of Plasma miR-126 for CAD Patients

**Figure 3:**
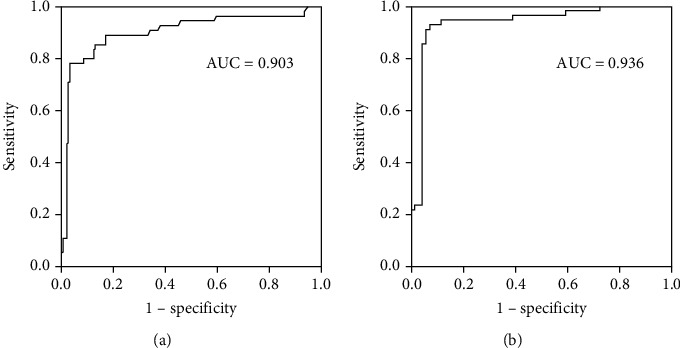
Diagnostic significance of plasma miR-126 for CAD subjects was evaluated by ROC curve analysis. (a) Healthy subjects versus stable CAD patients (AUC 0.903). (b) Healthy individuals versus unstable CAD subjects (AUC 0.936).

To investigate the clinical significance of circulatory miR-126 as a useful diagnostic biomarker of stable and unstable CAD, area under the Receiver Operating Characteristic (ROC) curve analyses were performed. ROC analysis showed that miR-126 strongly distinguished stable and unstable CAD subjects from healthy subjects with their high AUC predictive values were 0.903 (sensitivity 0.90%, specificity 0.95%) and 0.928 (sensitivity 0.92%, specificity 0.94%), respectively (highly significant with *P* < 0.001) (Figures 3(a) and 3(b)). Moreover, area under curve (AUC) values exhibited no significant correlation between stable and unstable CAD patients. These results indicated circulatory miR-126 may be considered a potential diagnostic biochemical marker for early prediction of CAD subjects.

### 3.5. Expression of miR-126 in HUVEC Cells and Luciferase Analysis

**Figure 4:**
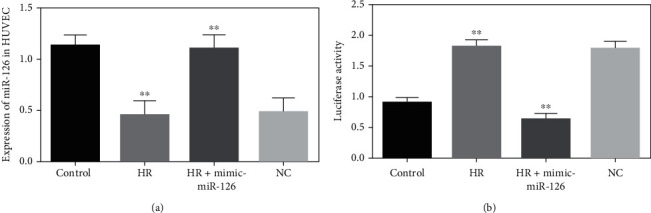
Expression of miR-126 in HUVECs and detection of the target gene. (a) Expression of miR-126 in normoxic, HR, HR transfected with mimic-miR-126, and negative control (NC) in HUVECs. (b) Luciferase reporter gene analysis among normal culture, HR, HR with mimic-miR-126, and NC groups in HUVECs and normalized against the expressions of Renilla reniformis. ^∗∗^*P* < 0.001.

HUVECs were exposed to 12-hour hypoxic and 6-hour normoxic conditions. The miR-126 expression levels were remarkably downregulated in H/R-injured HUVECs compared with normal HUVECs (*P* < 0.001). Conversely, miR-126 concentrations were markedly upregulated in H/R-exposed HUVECs treated with mimic-miR-126. However, no obvious differences were observed in miR-126 expressions between the H/R and negative control (NC) groups (Figure 4(a)). Moreover, luciferase activity levels were significantly elevated by 2-folds in H/R-incubated HUVEC (1.804 ± 0.11) cells than controls (0.903 ± 0.08), and there was no significant effect in negative controls. The luciferase expressions were remarkably regulated by the mimic-miR-126 in H/R-exposed HUVECs (Figure 4(b)).

### 3.6. Effects of Mimic-miR-126 on LRP6 and Cellular Viability in H/R-Exposed HUVEC Cells

**Figure 5:**
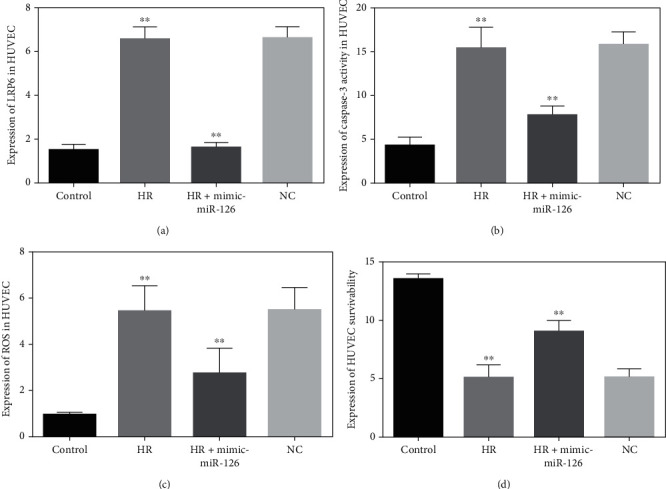
(a) LRP6 expression levels in normal, 18-hour HR, HR treated with mimic-miR-126, and NC groups in HUVECs. (b) Capase-3 expression between normal versus HR, HR versus HR plus mimic-miR-126, HR, and miR-126 NC groups in HUVECs. (c) Intracellular ROS concentrations in HUVECs between control and HR, HR, and HR transfected with mimic-miR-126, NC, and HR groups. ^∗∗^*P* < 0.001.

Compared with normoxic cells, the LRP6 levels were prominently upregulated in hypoxia/reoxygenation- (H/R-) incubated HUVECs which amazingly reversed back near to those of the control group upon transfection with mimic-miR-126. There was no effect observed between the H/R and negative control (NC) groups, indicating that miR-126 significantly regulated LRP6 expression levels in healthy and H/R-injured HUVECs (Figure 5(a)). Similarly, caspase-3 activities were highly elevated in H/R-induced HUVECs compared with normally cultured cells but, again, markedly reduced by transfecting H/R-injured HUVECs with mimic-miR-126. Among the NC group, there was no effect on caspase-3 activities (Figure 5(b)). The aforementioned findings strongly acknowledged that overexpression of miR-126 may exert a protective role against cellular damage in the H/R environment. The intracellular ROS concentrations were highly increased in H/R-induced HUVECs than those in controls. On the other hand, ROS levels in H/R-exposed HUVECs transfected with mimic-miR-126 were remarkably decreased compared with H/R groups, but ROS levels within NC and HR groups were not significantly changed, suggesting that mimic-miR-126 has major effects on preventing oxidative stress associated HUVEC cellular injuries during H/R conditions (Figure 5(c)). The HUVEC cellular viability was evidently reduced in H/R-exposed groups as compared with normal control groups. In contrast, overexpression of miR-126 by the mimic-miR-126 in H/R-cultured HUVEC group imparted a better outlook of cellular viability, while no clear changes were demonstrated in the HR and NC groups, suggesting that mimic-miR-126 may play an essential role in cellular protection against damage in H/R situations (Figure 5(d)).

## 4. Discussion

Atherosclerotic ischemic coronary heart disease is the single most important issue of premature death, and it imposes a major economic burden on the society across the world. Early identification and prompt appropriate management have a significant impact to reduce CAD mortality incidence. In the present study, we explored the clinical impact of miR-126 in CAD patients and its potential role to protect H/R-induced HUVECs cell damage. This study found that circulating miR-126 expression levels were evidently downregulated in stable and unstable CAD patients than healthy subjects, though miR-126 concentrations were comparatively lower in unstable angina groups than in stable groups, but the differences were not significant. Wang et al. demonstrated miR-126 expressions were obviously downregulated in the peripheral circulation of stable and unstable CAD patients compared with control subjects. Another study also reported expression of circulating miR-126 levels in single and multivessel CAD subjects was considerably lower than healthy subjects [9, 15].

The present research reported that miR-126 had significant correlation with CAD and aging. Circulating plasma miR-126 concentrations were evidently downregulated in geriatric (71-90 years) stable and unstable CAD patients of either sex as compared with comparatively younger age (30-50 years) patients. However, the expression patterns of peripheral circulatory miR-126 among healthy, stable, and unstable CAD groups did not show significant differences based on gender. Even among healthy volunteers, older male and female (71-90 years) participants had lower plasma miR-126 concentrations than the younger (30-50 years) did, but statistically nonsignificant. Very recently, Alessia et al. demonstrated miR-126 has linked with Fabry disease (FD) and premature aging [16]. Another study showed that in the middle age type-2 diabetic patients, senescence was associated with reduced miR-126 levels [17].

Diagnostic significance of circulatory miR-126 levels was determined through ROC curve analysis. The CAD patients, either stable or unstable, were strongly demarcated from healthy subjects with high sensitivity and specificity and AUC of 0.903 and 0.936, respectively, and that was highly significant statistically. However, utilization of circulatory miR-126 levels in differentiation between stable and unstable CAD groups was nonsignificant. These results indicated downregulated plasma miR-126 might serve as a useful noninvasive biomarker for early evaluation of CAD patients which is supported by other clinical studies [9, 15]. This study demonstrated hs-CRP concentrations being significantly higher in unstable and stable CAD patients in reference to the healthy participants. Our prior study also reported that elevated hs-CRP is linked with coronary artery disease and atherosclerosis which requires more molecular studies to discover the underlying mechanisms. Worth mentioning, family history was found to have a strong association with CAD [18].

It has been well known that mutation or dysregulation of LRP6 proteins is directly associated with oxidative stress, dyslipidemia, atherosclerosis, and coronary artery disease [19–21]. Caspase-3 activities were enormously increased in 18-hour H/R-exposed HUVECs but remarkably reduced after transfection with mimic-126. Similarly, the intracellular ROS levels in H/R-stressed HUVECs were highly elevated, but markedly reduced after being incubated with mimic-miR-126. Furthermore, cellular viability noticeably decreased in H/R-HUVECs as compared with normal cells and exceptionally increased after transfection with mimic-miR-126. These findings suggested that mimic-miR-126 protected against H/R-induced cellular injuries; hence, it could be a useful therapeutic target for atherosclerotic coronary artery disease. However, this is a single-center, tertiary-level hospital-based study with relatively small sample size. Therefore, multicentric larger clinical studies will be required to confirm the positive role miR-126 as a biomarker and promising therapeutic target in ischemic heart disease.

## 5. Conclusion

Downregulated plasma miR-126 levels may be able to serve as a new biomarker for early diagnosis of stable and unstable CAD patients, and mimic-miR-126 could be a potential therapeutic target in atherosclerotic coronary artery disease by inhibiting LRP6 protein expression.

## Figures and Tables

**Table 1:** Clinical data of the study population.

Variables	Healthy volunteers (55)	Stable CAD group (153)	Unstable CAD group (70)	*P*_1_	*P*_2_	*P*_3_
Age (years)	57.2 ± 11.3	65.1 ± 12.4	68.5 ± 14.8	0.186	0.157	0.319
Male/female	30/25	85/68	43/27	0.122	0.504	0.183
Current smoker	59% (32)	72% (110)	75% (52)	0.708	0.643	1.00
Essential hypertension	—	74% (113)	82% (57)	—	—	0.068
Hyperlipidemia	—	85% (130)	89% (62)	—	—	0.274
Family history	9% (5)	63% (96)	66% (46)	0.000	0.000	0.832
Diabetes mellitus	—	26% (40)	32% (22)	—	—	0.159
SBP (mmHg)	122.2 ± 10.3	129.4 ± 14.2	132.8 ± 12.7	0.085	0.066	0.114
DBP (mmHg)	74.6 ± 6.8	80.5 ± 7.3	82.1 ± 9.5	0.094	0.081	0.132
Heart rate (beats/min)	71.9 ± 8.6	75.2 ± 10.3	78.4 ± 11.2	0.407	0.375	0.729
Fasting glucose (mmol/L)	4.79 ± 0.5	5.06 ± 0.9	5.3 ± 1.2	0.073	0.069	0.251
Cr (*μ*mol/L)	80.9 ± 18.1	82.5 ± 22.7	85.6 ± 24.8	0.073	0.069	0.148
hs-CRP (mg/L)	2.7 ± 1.68	11.3 ± 9.7	16.5 ± 11.4	0.000	0.000	0.091
Total cholesterol	4.2 ± 0.73	4.39 ± 0.75	4.56 ± 1.3	0.084	0.075	0.116
HDL-C (mmol/L)	1.1 ± 0.4	0.92 ± 0.6	0.83 ± 0.7	0.095	0.087	0.123
Triglyceride (mmol/L)	1.26 ± 0.5	1.39 ± 0.9	1.48 ± 0.7	0.099	0.095	0.104
LDL-C (mmol/L)	2.21 ± 1.3	2.44 ± 1.7	2.51 ± 1.6	0.219	0.157	0.289
Left ventricular ejection fraction (%)	61.7 ± 9.4	58.7 ± 10.3	54.2 ± 10.7	0.326	0.271	0.467
AST (U/L)	11 ± 7.6	13 ± 9.3	16 ± 7.1	0.794	0.592	0.813
ALT (U/L)	14 ± 5.6	18 ± 8.2	20 ± 7.6	0.203	0.177	0.369

CAD: coronary artery disease patients; SBP: systolic blood pressure; DBP: diastolic blood pressure; AST: aspartate aminotransferase; ALT: alanine aminotransferase; *P*_1_: healthy participants and stable CAD; *P*_2_: health control subject versus unstable CAD; *P*_3_: stable CAD versus unstable CAD patients.

## Data Availability

From corresponding author upon reasonable request data will be available.

## References

[1] Virani S. S., Alonso A., Aparicio H. J. (2021). Heart disease and stroke Statistics-2021 update: a report from the American Heart Association. *Circulation*.

[2] Lu Y., Thavarajah T., Gu W., Cai J., Xu Q. (2018). Impact of miRNA in atherosclerosis. *Arteriosclerosis, Thrombosis, and Vascular Biology*.

[3] Churov A., Summerhill V., Grechko A., Orekhova V., Orekhov A. (2019). MicroRNAs as potential biomarkers in atherosclerosis. *International Journal of Molecular Sciences*.

[4] Yan Y., Song D., Wu J., Wang J. (2020). Long non-coding RNAs link oxidized low-density lipoprotein with the inflammatory response of macrophages in atherogenesis. *Frontiers in Immunology*.

[5] Hao X.-Z., Fan H.-M. (2017). Identification of miRNAs as atherosclerosis biomarkers and functional role of miR-126 in atherosclerosis progression through MAPK signalling pathway. *European Review for Medical and Pharmacological Sciences*.

[6] Kang S. (2020). Low-density lipoprotein receptor-related protein 6-mediated signaling pathways and associated cardiovascular diseases: diagnostic and therapeutic opportunities. *Human Genetics*.

[7] Keramati A. R., Singh R., Lin A. (2011). Wild-type LRP6 inhibits, whereas atherosclerosis-linked LRP6R611C increases PDGF-dependent vascular smooth muscle cell proliferation. *Proc Natl Acad Sci USA.*.

[8] Xu Y., Gong W., Peng J. (2014). Functional analysis LRP6 novel mutations in patients with coronary artery disease. *PLoS One*.

[9] Li H.-Y., Zhao X., Liu Y.-Z. (2016). Plasma microRNA-126-5p is associated with the complexity and severity of coronary artery disease in patients with stable angina pectoris. *Cellular Physiology and Biochemistry*.

[10] Ling H., Guo Z., Shi Y., Zhang L., Song C. (2020). Serum exosomal microRNA-21, microRNA-126, and PTEN are novel biomarkers for diagnosis of acute coronary syndrome. *Frontiers in Physiology*.

[11] Yang H.-H., Chen Y., Gao C.-Y., Cui Z.-T., Yao J.-M. (2017). Protective effects of microRNA-126 on human cardiac microvascular endothelial cells against hypoxia/reoxygenation-induced injury and inflammatory response by activating PI3K/Akt/eNOS signaling pathway. *Cellular Physiology and Biochemistry*.

[12] Alique M., Bodega G., Giannarelli C., Carracedo J., Ramírez R. (2019). MicroRNA-126 regulates hypoxia-inducible factor-1*α* which inhibited migration, proliferation, and angiogenesis in replicative endothelial senescence. *Scientific Reports*.

[13] Sheikh M. S. A. (2020). Diagnostic role of plasma microRNA-21 in stable and unstable angina patients and association with aging. *Cardiology Research and Practice*.

[14] Ali Sheikh M. S., Salma U., Zhang B., Chen J., Zhuang J., Ping Z. (2016). Diagnostic, prognostic, and therapeutic value of circulating miRNAs in heart failure patients associated with oxidative stress. *Oxidative Medicine and Cellular Longevity*.

[15] Wang X., Lian Y., Wen X. (2017). Expression of miR-126 and its potential function in coronary artery disease. *African Health Sciences*.

[16] Lo Curto A., Taverna S., Costa M. A. (2021). Can be miR-126-3p a biomarker of premature aging? An ex vivo and in vitro study in Fabry disease. *Cell*.

[17] Banerjee J., Roy S., Dhas Y., Mishra N. (2020). Senescence-associated miR-34a and miR-126 in middle-aged Indians with type 2 diabetes. *Clinical and Experimental Medicine*.

[18] Sheikh M. S. A. (2021). Role of plasma soluble lectin-like oxidized low-density lipoprotein receptor-1 and microRNA-98 in severity and risk of coronary artery disease. *Balkan Medical Journal*.

[19] Jeong W., Jho E.-h. (2021). Regulation of the low-density lipoprotein receptor-related protein LRP6 and its association with disease: Wnt/*β*-catenin signaling and beyond. *Frontiers in Cell and Development Biology*.

[20] Çakmak H. A., Demir M. (2020). MicroRNA and cardiovascular diseases. *Balkan Medical Journal*.

[21] Jansen F., Stumpf T., Proebsting S. (2017). Intercellular transfer of miR-126-3p by endothelial microparticles reduces vascular smooth muscle cell proliferation and limits neointima formation by inhibiting LRP6. *Journal of Molecular and Cellular Cardiology*.

